# FEMALE BREAST CANCER SURVIVAL IN BLACKS VERSUS WHITES ACCORDING TO TUMOR STAGE, AGE, AND MARITAL STATUS AT DIAGNOSIS

**DOI:** 10.1093/geroni/igad104.3355

**Published:** 2023-12-21

**Authors:** Ray Merrill, Ian Gibbons

**Affiliations:** Brigham Young University, Provo, Utah, United States; Brigham Young University, Provo, Utah, United States

## Abstract

Female breast cancer survival is significantly influenced by tumor stage, age, and marital status at diagnosis. While Blacks have historically had lower breast cancer survival than Whites, it is not clear whether this lower survival differs across the levels of tumor stage, age, or marital status at diagnosis. This paper will assess this using five-year relative survival (5-YRS) estimates from the Surveillance, Epidemiology, and End Results (SEER) Program. Cases reflect 2000-2015 and follow-up extends through 2020. 5-YRS was 90.9% in Whites and 81.1% in Blacks, consistently lower by the levels of tumor stage, age, and marital status at diagnosis. In a regression model adjusting for these variables, the difference in 5-YRS was 8.0% for Whites versus Blacks, -15% for regional stage and -67% for distant stage (versus local stage), lower in older ages at diagnosis, and 5.2% for married (or cohabitating) versus otherwise. The lower 5-YRS in Blacks significantly varied by tumor stage at diagnosis (4.2% for local stage, 9.9% for regional stage, and 11.6% for distant stage; p=0.0098) but did not significantly vary by age or marital status at diagnosis. The variation across tumor stage at diagnosis in the lower 5-YRS for Blacks depended on age (p< 0.0001), with greater variability in the younger age groups and less variability in the older age groups. Thus, poorer 5-YRS for Blacks versus Whites persists across tumor stage, age, and marital status at diagnosis and becomes increasingly more pronounced in later stages at diagnosis, especially in younger versus older age at diagnosis.

